# Detecting and Tracking Criminals in the Real World through an IoT-Based System

**DOI:** 10.3390/s20133795

**Published:** 2020-07-07

**Authors:** Andrea Tundis, Humayun Kaleem, Max Mühlhäuser

**Affiliations:** 1 Department of Computer Science, Technische Universität Darmstadt, 64289 Darmstadt, Germany; max@tk.tu-darmstadt.de; 2 Fraunhofer SIT, Institute for Secure Information Technology, 64295 Darmstadt, Germany; humayun.k.khan@outlook.com

**Keywords:** IoT, safety, smart city, simulation, crime detection, crime tracking, terrorism

## Abstract

Criminals and related illegal activities represent problems that are neither trivial to predict nor easy to handle once they are identified. The Police Forces (PFs) typically base their strategies solely on their intra-communication, by neglecting the involvement of third parties, such as the citizens, in the investigation chain which results in a lack of timeliness among the occurrence of the criminal event, its identification, and intervention. In this regard, a system based on IoT social devices, for supporting the detection and tracking of criminals in the real world, is proposed. It aims to enable the communication and collaboration between citizens and PFs in the criminal investigation process by combining app-based technologies and embracing the advantages of an Edge-based architecture in terms of responsiveness, energy saving, local data computation, and distribution, along with information sharing. The proposed model as well as the algorithms, defined on the top of it, have been evaluated through a simulator for showing the logic of the system functioning, whereas the functionality of the app was assessed through a user study conducted upon a group of 30 users. Finally, the additional advantage in terms of intervention time was compared to statistical results.

## 1. Introduction

Terrorist and criminal events are phenomena that have always plagued human society. Lately, they have become increasingly frequent at European level, such as have recently occurred in Paris, in Nice, and in Berlin [1]. Unfortunately, despite of the efforts made by European governments in collaboration with Law Enforcement Agencies (LEAs), for example, by increasing the presence of human resources during specific public events in specific places, these measures are not always effective to deal with such problems, due to the level of fine granularity, both from the temporal and spatial point of view, with which a criminal event can take place. In fact, it makes it practically unfeasible to predict where, when, and how a crime takes place, in order to avoid it or to be able to react promptly when it occurs. LEAs and Emergency Services (ESs) struggle to protect and help people in need, partially because their structural organization is not flexible enough. Moreover, there is a lack of communication means of the Police Forces (PFs), who do not actually have an effective communication system with the citizens, that is “common men” or “common people”, who represent the direct victims of crimes. In the first place, one should be aware of, that reducing, if not eliminating, crimes is not only a problem of the governments but of the entire society and, in particular, it is directly associated with citizens, whose involvement has not been fully considered in the investigation process yet. This is one of the main objectives of this work, which is an extended version of [2]. It was part of a EU H2020 research project called TAKEDOWN [3], that was focused on the understanding of Organized crimes and Terrorism phenomena with the aim to deliver cyber solutions by focusing on an advanced collaboration among citizens and LEAs.

In general, cities are exploring innovative approaches to deal with emergency situations [4]. Security agencies are turning towards Information Technology (IT) [5,6], as well as by improving the intelligence systems through the collection of data, to solve cases and reduce the crime rate or even capture criminals [7]. In recent times, the increase in processing power and the use of social IoT devices (e.g., smart-phones and -watches [8]) have made them prone to abuse. On the other hand, they have become a strong crime-solving and crime-fighting tools thanks to the advancement of 3G/4G cellular networks [9], for example, by reporting of incidents via mobile phones [10] as well as by supporting crime detection [11,12].

Indeed, the exploitation of smartphone devices in combination with a layered computation and communication-based mechanism may represent a potential approach to improve people’s safety, without being too invasive into their privacy by using a minimal set of data. In fact, due to the growing diffusion of connected mobile devices mediated by the network, it is possible to establish collaborations, share data, provide real-time information on specific events, as well as to perform on-site computation by moving towards “Smarter Cities” [13]. As a consequence, instead of being only a victim of a criminal event, a more active role can be played by the citizens not only by directly communicating with the PFs, but also among each other. Indeed, they can provide timely and distributed information that, according to the Edge computing paradigm, can be exploited for local computation in order to support the decision making process at local/regional level [14].

In this direction, the paper proposes an IoT-based system, which aims at enhancing the communication between citizens and LEAs. It is centered on smartphones’ devices and relies on an edge architectural approach. Two main devices’ features are exploited: timestamps and location. They are employed to enable the criminal detection in the real world through the generation of crime reports related to time and place of specific crime spots in real-time, to track the evolution of criminal-related events, as well as to predict potential directions of a criminal. Both concepts and features, along with the proposed algorithms, have been defined in collaboration with the PFs, and in particular with Valencia Local Police (Spain), on the basis of their experience and needs. Indeed, the generated information can be used by them: (i) on one hand, in order to know in advance the movements of the criminal, and therefore from a logistical point of view, for organizing and implementing specific countermeasures, and (ii) on the other hand, to improve citizens’ safety through a real-time information service based on messages. The system’s logic has been evaluated through the use of simulation techniques. In particular, the system provides a simulated operating mode, in which the role of citizens and criminals is replaced and emulated by virtual citizens and virtual criminals. This simulation environment has been used not only to evaluate the behavior and operation of the current proposed algorithms, but it allows also to support the definition and evaluation of more sophisticated additional algorithms. Whereas, its real functioning based on apps installed on mobile devices has been evaluated through (i) a case study with 30 participants, in order to assess the solution from the user perspective, as well as (ii) a qualitative analysis to estimate its effectiveness has been conducted by estimating time intervention and time saving in big cities in comparison to real statistical data.

The rest of the paper is organized as follows. Related works on tracking of criminals and crimes, along with a background on exploited basics concepts, are discussed in Section 2. In Section 3, a description of a typical criminal scenario along with the tackled research questions are described. The proposed solution is elaborated in Section 4, whereas the implementation details of the system and its functioning is shown in Section 5. Section 6 concludes the paper by summarizing the contribution and sketching future research directions.

## 2. Related Work and Background

An overview on Internet of Things (IoTs), Mobile Apps, as well as Collaborative Systems (CSs), which represent the theoretical key aspects on which this research work is based, is given in Section 2.1. Then, a summary of research efforts on crime and emergency situations is provided in Section 2.2, whereas a comparison of the available Apps is reported in Section 2.3.

### 2.1. Concepts of Iot, Collaborative Systems, and Mobile Apps

The term *Internet of Things (IoTs)* [15] became popular when the importance of the Radio-Frequency IDentification (RFID), as an enabling technology for the IoTs that would have allowed computers to manage individual things, was understood [14]. IoTs can be described as a system of interrelated computing devices, objects, animals, or people that are provided with unique identifiers and able to transfer data over a network without requiring human-to-human or human-to-computer interaction [16]. Another term which is also common nowadays is the Internet of Things devices [17], that is, non-standard computing devices that connect wirelessly to a network and which have the ability to transmit data. It includes smartphones or any device which is capable of communicating and interacting over the Internet and which can be remotely monitored and controlled.

On the other hand, a *Collaborative System* is an information system used to facilitate efficient sharing of data, documents, files, information, and knowledge between different entities, which have a common goal in order to achieve a specific objective [18]. Thanks to the advent of smart devices, more advanced and flexible forms of collaborative systems have emerged, which are called Mobile Collaborative Systems (MCS) [19]. Each entity (i.e., mobile unit) plays a different role, and the decision of the system, in solving the task, depends from each single information coming from the involved entities.

A *Mobile App* is, instead, a software application designed to run on mobile devices such as smartphones, smartwatches, or tablets. Some of the main features of mobile apps relies on Social Integration, Customization, Push Notifications, Augmented Reality, Feedback System, Advanced Analytics, Payment Gateway Integration, and so on. Furthermore, GPS and location-based services are the most popular, as they enabled specific geolocation-based services [20,21].

### 2.2. Research Paper

In [22], a solution to improve the user safety based on mobile devices, in order to notify the citizens regarding crime prone areas, the nearest Police Station, and community precincts for assistance in case of emergency, is provided. It consists of two components: (i) the server side which is accessible to the police, for keeping the system updated with past crime events committed within a specific area of the city, crime rate, and so on; (ii) the user side, which is accessible to citizens. On the basis of the geolocation, the information regarding the top dangerous streets of the city, safety recommendations, the closest police station, and public transportation services is provided.

Instead, a web-based criminal record system (CRS), to support the police to record the location of crimes using location-based services embedded in the mobile devices, is proposed in [23]. This solution allows the user to use, additionally, the camera as well as the sound system to enrich the location-related data with visual data and sounds. The full crime record is then used to support further analyze such as studying the distribution of crimes in different geographic areas by creating crime mapping.

The work described in [24] introduces an emergency response application, which combines both mobile and web applications in order to promptly react to the requests of ambulances, fireman, or police in situations of emergency. In particular, the mobile application aims to quickly detect the location of the user, who generates the request, using the geolocation and to send it to the web application, which represents the brain of the system, in order to support the decisions for dispatching of emergency units.

In [25], a study to identify both limitations and main features regarding applications related to emergency responses was conducted. The main objective was to identify specific aspects able to drive the design phases of mobile emergency applications, by analyzing already existing contributions as well as by directly experiencing the development and the evaluation of solutions related to emergency situations. The sensors available in the mobile devices have been exploited by studying the users’ behavior for using them, whereas the evaluation approach was based on (i) a practical part, where an emergency scenario related to a traffic accident has been simulated, with the victim lying close to the car in the middle of the street, and (ii) a theoretical part, conducted through a survey where experts in emergency situations have been interviewed, so as to evaluate qualitatively the utility of the proposed solution.

Whereas, in [26] a mobile application for the Philippine National Police Emergency Hotline is proposed. It aimed to make faster the response time by reducing the time to collect and elaborate the date during a phone call (e.g., personal information). This was done through user profile registration to be able to use the app. The data of the user becomes visible to the dispatcher if a help is requested. Once a request takes place, such sensitive personal data, along with those regarding the surrounding environment of the caller, are visible to the responders in order to proceed with the help.

In [27], a Social Video Streaming (SVS) application that uses smart phones for street crime reporting by recording the video of incidents is proposed. This application is meant to be used by the citizens when they come across an incident such as a robbery or any crime incident that is happening in their surroundings. By using SVS application, they can send live video to the recording server managed by the police authority in order to helping them to get information on the location of the crime. In [4], instead, the design of crime detection system centered on IoT devices, which is meant to support the detection of crimes in real-time based on the human emotions, is introduced. Here, the authors tried to identify whether a user is in dangerous, on the basis of wearable sensing device attached to the user’s underwear.

A summary of the above-described related works is reported in Table 1.

### 2.3. Emergency-Related Apps

In this section, the most common App-based security services enabled by social devices, and in particular by smartphones, are below presented and compared in Table 2. They can be grouped in four main categories: *Short-Cut*, *Emergency-Information*, *Crime/Disaster-Tracking*, and *Geo-Fencing*.

*Short-Cut Apps* aim to provide the user with fast access to more complex functions, mainly different ways of triggering emergency calls while barely needing user interactions. An example is represented by “Red Panic Button” created by ”Ultimate Communication Software LTD” [28], which is available for Android and Apple devices. The core function is represented by a single red button. When it is pushed, depending on the configuration, a panic sms or an email, containing the location of the user, is sent out. This is typically useful in situation of illness. Another app, called “SafeTrek” [29], triggers an emergency message to the police, instead of an emergency call, on the release of a button, if the user does not enter a PIN code shortly after. Another tool is represented by “bSafe” [30], which is an app configurable in terms of name, location, audio, and video to be communicated.

*Emergency-Information Apps* are pretty simple and loosely connected to the first type. The purpose of these kinds of apps is to provide the health information of the user to the first responder. This category was populated by a wide variety of apps, so that Android, Apple, and Google decided to add this functionality directly to their smartphone operation systems [31,32].

*Crime/Disaster-Tracking Apps* have very straightforward functions and purposes. Depending on the specific type of app, it can be simply related to crime or disaster statistics for that area or some apps can also directly provide warnings of ongoing (or approaching) threats. For example, “Citizen” is an app that was created by “Sp0n Inc.” [33], which works by aggregating information about emergencies and ongoing crimes from the police and its users mainly via a live chat. “Warn-App NINA” is, instead, a German application, which is mainly used by the government to communicate with the citizens and inform them about already existing disasters or dangerous situations, such as major fires or hazardous substances spreading in an uncontrolled manner and so on [34]. “Crime Mapping” is an app created by “TriTech Software Systems” [35] which collects and aggregates crime data mostly provided by LEAs. After that the user selects an area, available crime data and statistics are displayed. “Disaster Alert” is an app created by the “Pacific Disaster Center” [36] which collects disaster and hazard information from different agencies and services all around the world and displays them on a world map or in a chronological list. Furthermore, as in the last few years live streaming technology has become very popular and more accessible to people, a proliferation of different mobile applications, such as “Ustream”, is used from the citizens for street crime reporting, by exploiting the live streaming functionality [27].

*Geo-Fencing Apps* refers the creation of a virtual perimeter, based on real-world coordinates, which can be used to trigger certain actions depending on a user or object location. “Safety Guardian” is one of those apps [37] which allows the users to create geo-fences and apply triggers to them for either joining or leaving their defined areas. The app is able to trigger either notifications, SMS, or phone calls. The application requires a download from google maps and a pre-configuration of fences and triggers by the user. It can be even used for a trivial safety reminder with its notifications, for example, by reminding the user to turn of the stove after leaving the house, a more serious safety reminder such as warnings before entering a dangerous area. “Watch Over Me” [38] is instead an application that lets the user select emergency contact list to be contacted if the user fail to check-in safely at your destination. The app monitors the user’s program and related path (e.g., run, walking, taking a taxi, or meeting someone) via GPS. If he fails to check-in safely into destination, the app will alert his emergency contacts with his location. The application has features such as shake your phone to trigger an emergency alert and also activates your phone’s camera to record what is happening. It also triggers a notification if the user is in a high crime rate area to make you more cautious. A similar application is LifeLine Response [39] which also supports wearables like Apple Watch and Garmin. It provides extra functions such as if the thumb leaves the phone’s screen in case of attacker snatching the phone and if the user is unable to enter the disarm code, an alert is sent to the authorities, meanwhile the phone will be emitting an ear-piercing sound while flashing, while authorities are en route. In the event of phone being broken, the app notices the connection being dropped and sends the user’s latest location to the authorities.

Other research activities and projects, which are not reported in Table 2, are focusing on emergency situations. One of those efforts is represented by the Guardian Project which defines the PanicKit Framework [40] for letting panic trigger and receiver apps connect with each other. Another result is represented by the AppArmor–Safety Platform [41] which provides features related the previously described app categories as well as additional location related information, such as emergency plans.

## 3. Main Motivations

This section provides an overview of the main problem under consideration and its related aspects, as it is highlighted in Section 3.1, as well as the emerged research questions, which are discussed in Section 3.2, that have been tackled.

### 3.1. Problem Statement

**Figure 1:**
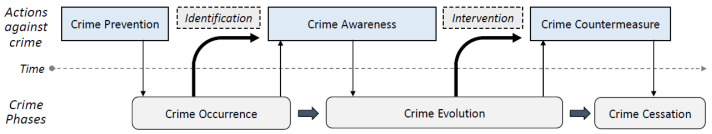
Life cycle of a criminal scenario and actions against it.

The fight against criminal activities has been always one of the most complicated challenges to be faced due to multiple factors [42,43,44]. In fact, the occurrence of a crime can (i) take place because of political, economic, religious, or personal reasons; (ii) happen in the most diverse parts of the world; and (iii) be conducted with a granularity ranging from a single person or group of them, up to widespread criminal organizations. However, all types of crimes, independently of their nature and granularity, share some important aspects that characterize a “criminal scenario”. According to our vision the life cycle of a “criminal scenario” can be broken down in three main phases, which are depicted in Figure 1: *crime occurrence*, *crime evolution*, and *crime cessation*. In particular, (i) *crime occurrence* represents the period of time when a crime starts, that is to say, when an event is perceived from the external as threat; (ii) *crime evolution* represents the subsequent of actions that take place after that a crime event occurs. It can, in turn, keep on representing a threat for the people in the surrounding environment; (iii) *crime cessation* is the moment or time interval from which the crime event does not affect anymore the environment or, it does not threat anymore the safety of other individuals in the surroundings.

Beside that, different actions against crimes can be adopted in various instants of the life cycle of a criminal scenario:

- *Crime prevention*, that is related to all efforts made by the LEAs and PFs to cope with crime events by taking all the preliminary measures by attempting to reduce and deter crimes and criminals.
- *Crime awareness*, which takes place when the PFs discover an ongoing crime, meaning that in a specific time period, they are aware of what is happening and where it is happening.
- *Crime countermeasure*, that is a measure or action taken to counter or compensate another one. It can be represented by any technological or tactical solution or system, that is designed to prevent an undesirable outcome in the process. In this case, it is related to everything that can be done to stop the crime.

Figure 1 shows the temporal relationships between crime phases and the actions to deal with them. In this perspective, the main weak links rely on the *Identification* and *Intervention* time due to lacks in terms of communication and coordination timeliness because (i) LEAs and PFs are usually not in the place, where and when a crime event or an attack takes place; (ii) the time between the occurrence of a crime event, its notification to the LEAs (i.e., *Crime Awareness*) and their arrival on the place, is typically too long to be able to apply effective countermeasures in time. As a consequence, few minutes (or seconds) delay can have a strong impact on the surrounding environment and be even crucial for many citizens’ life.

### 3.2. Research Questions

In the light of the above discussed motivations, the following four research questions have been tackled in this paper.

Question 1—How can the time between the occurrence of a crime/attack and the awareness of the PFs be reduced?

In principle, the occurrence of a crime event can take place anytime and anywhere. As a consequence, law enforcement and police forces are highly unlikely to be present at the crime scene at the time of the crime, except for specific reasons such as public events or ufficial ceremonies and celebrations and so on. In case of non-presence at the crime scene, the first problem lies in the timing with which the PFs are aware of such event. Typically, the information comes from the citizens, (i) through a domino effect communication as long as it reaches, by chance or randomly, the law enforcement, or (ii) after overcoming the state of panic by directly communicating the ongoing crime event by phone call. From one side, this usually takes a while and even on the call it is difficult to exactly communicate the location of the criminal to the police. As a consequence, a support for obtaining, selecting, grouping, and processing data related to crime events by distinguishing them for location represents a primary need.

Question 2—How can the evolution of a crime and of a criminal be tracked and reported in real-time to the local LEAs/PFs?

After the crime scene is identified, it is highly unlikely that the criminal stays there for a long time. As a consequence, not only the criminal can move, but also the criminal activity evolves accordingly, thus making it even more difficult to be intercepted. This means that not only the law enforcement officers arrive with some delay at the initial crime scene, but both the criminal may no longer be there, and probably continuing to commit crimes elsewhere. This entails additional time both to identify again the new crime scene and to reach it, which could repeat over the time. In this case timeliness represents an important factor to be considered. Consequently, the computation of new additional data, in order to derive real-time useful information, requires timely processing along with reduced network latency times.

Question 3—How can the movement of a criminal be predicted?

Another additional aspect regarding the analysis of the crime dynamics concerns the understanding of the criminal’s moves in advance. After starting a crime, for example by shooting in a crowd in a square, the criminal could be willing to continue this activity by heading to another crowded place, such as to a club or hotel, etc. On the contrary, after committing the crime, the criminal could be willing to run away and escape, for example by going to the train station. In this case, predicting a particular movement, towards a potential direction or a place to which the criminal might be directed, would be of paramount importance from the strategic point of view for the police to intervene and block the criminal with a strong impact on the human safety.

Question 4—How can the local safety of citizens be increased?

The last but not least aspect regards the information level provided to the citizens related to the ongoing danger situation. Even though the citizens represent the first victims of a crime, those who are close to the crime scene do not get to know about the ongoing situation unless, in the instant of time in which they are involved in it, and therefore become part of the crime as victims. Having the opportunity to get to know about a specific danger in progress in the surrounding area and in real-time would have an important impact on the general safety of the citizens, for example, by receiving specific precautionary information, or guidelines on how to behave, where to go, how to avoid the danger, and so on.

## 4. An Iot-Based Communication Model for Criminals Detection and Tracking

In this section, an IoT-based mobile collaboration system for detecting and tracking criminals in the real world is elaborated. First, an edge-based architecture and its main advantages are highlighted, and then the proposed key concepts (i.e., *crime report (cr), tracking point (tp), tracking path (tpa), path prediction (pp)*, and *back notification (bn)*) and the algorithms, which are defined on the top of them, are described.

### 4.1. Edge-Based Architecture and Related Advantages

**Figure 2:**
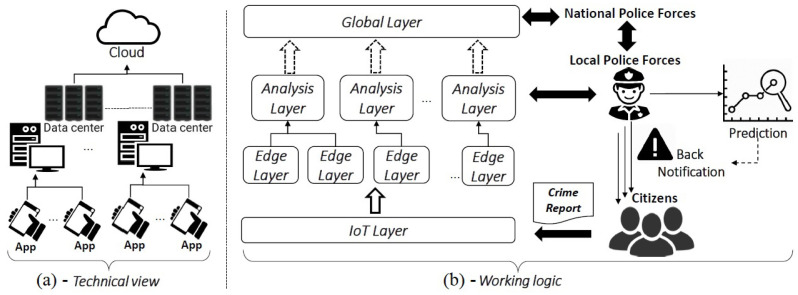
Edge-based architectural model.

Figure 2b provides an overview of the architectural model by showing its system operation, the involved actors, and their role. It is based on an hybrid version of “The Regional Edge” and “The Access Edge” edge infrastructure phases, whose concepts are discussed in [45]. In particular, (i) “The Regional Edge” extends the edge a layer deeper, with the aims of leveraging the infrastructure in hundreds or thousands of locations instead of hyperscale data centers in a few sites, and (ii) “The Access Edge” is characterized by a micro-data center, whose size and computing power can be adjusted according to local needs. Starting from the above concepts, four computational layers have been proposed in this work for the dataflow management: *IoT layer*, *Edge layer*, *Analysis layer*, and *Global layer*.

- *IoT layer*. It is the layer through which the information about a crime event is exchanged among citizens and PFs and it deals with questions 1 and 4 presented in Section 3.2. The fundamental role is played by the citizens, who can report data, by using IoT social devices, related to an event and its dynamic. The data gathered from the citizens is used to elaborate specific responses and emergency countermeasures related to the crime into account.
- *Edge layer*. This local computational layer, which deals with questions 2 and 3 presented in Section 3.2, aims at computing the rough local data coming from the IoT layer. In particular, the data which comes from different sources is selected, organized, and grouped on a regional basis, according to specific principles of locality, in order to derive and generate additional information. The advantage of this layer relies in the timeliness in obtaining useful information centered on local and recent data.
- *Analysis layer*. It tackles questions 3 and 4 presented in Section 3.2. At this level the information coming from the *Edge Layer* is further elaborated from a local “micro data center”, to support the decision of the local Police Forces (PFs) related to a specific area for dealing with the criminals. Indeed, local views can be obtained from the local PFs based on their area. Such local data can be then used (i) to predict potential movements and directions of the criminal and the related evolution of the criminal events, as well as (ii) to enhance the safety of the citizens by providing them back notifications and real-time safety suggestions about the ongoing crime event. The main advantage relies in the automatic routing and information redistribution to the citizens in a decentralized way.
- *Global layer*. The data coming from the local *Analysis Layer(s)* can be globally elaborated in Cloud, from the National Police Forces (NPFs), to perform further analysis and statistics, in order to make offline decisions.

From a technical point of view, different strategies for deploying edge platforms are available, as described for example in [46]. The proposed layered approach is independent from the technical perspective, however to give an idea of a possible realization, a viable implementation is depicted in Figure 2a, which also highlights the relations among the 4-layer model and its technical details.

It worth noting that no particular innovation is tied to the pure edge computing field, but in this case, the “Edge” plays the role of enabling technologies in the context of criminal detection and tracking, due to its advantages. Indeed, the strength of the edge-based architecture relies on the use of IoT devices which are located at the network edge, by enabling a pervasive and ubiquitous communication and collaboration between citizens and PFs. In addition, the system has advantages in terms of (i) energy savings due to less communication, (ii) greater responsiveness as the processing is partially decentralized, (iii) more privacy preserving as the data remains locally, and (iv) less bandwidth usage, as the data is first locally preprocessed and then sent.

### 4.2. Crime Report

A *crime report (cr)* aims to deal with the first question discussed in Section 3. It is meant to be provided by the citizens, as they are the closest actors during a crime scene and they are the ones who can report at earliest such event. It is modeled through the set of features, which are reported in Table 3. They allow to express the occurrence of a crime, on the basis of data provided by the citizen (i.e., the user) who reports the crime. In particular, the data related to the crime location is derived starting from the *User Coordinates (uc)*, whereas the temporal occurrence of a crime is elaborated on the basis of the user’s *(Report) Timestamp (ts)*. Additional data can be used to better characterize a specific crime, if available. For example, the most common 15 predefined *Crime Types (ct)*, identified in cooperation with the Valencia Local Police (Spain) on the basis of their statistics, as well as an unstructured *Textual Description (td)*, can be optionally provided.

Equation (1) represents a model of a *crime report (cr)* by using the above described features centered on the user data, whereas the pseudocode, that has defined on the top of them, is reported through the Algorithm 1. It shows how the above mentioned features are extracted from an *UserData u* provided by a user/citizen, in order to generate a crime report cr.

(1) $$CrimeReport\left(cr\right)=<lo,ts,ct^{+},td^{+}>$$

**Algorithm 1:** Generation of a Crime Report

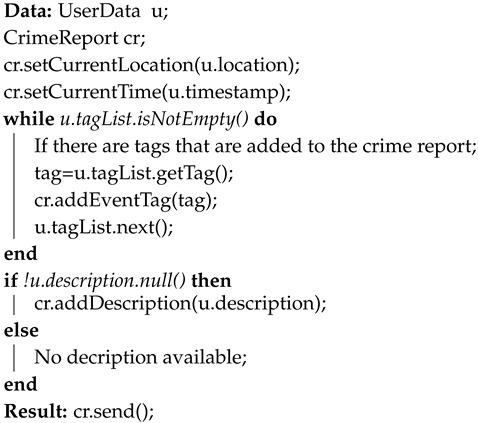



### 4.3. Tracking Point and Tracking Path

A *tracking point (tp)* is an information derived from one or more crime reports, which is used to observe the evolution of criminal events. As a consequence, given a crime report $cr^{e}$ related to a crime event *e*, a tracking point $tp^{e}$, which relies at least on $cr^{e}$, aims to keep on tracking such event *e* on the basis of the data obtained through its associated crime report $cr^{e}$.

By generalizing, given a set of already existing crime reports associated to the event *e*, $CR_{e}=\left\{cr_{1}^{e},cr_{2}^{e},\ldots ,cr_{i}^{e},\ldots ,cr_{n}^{e}\right\}$, with i=1,…,n, a tracking point $tp_{n+1}^{e}$ is computed, when a $cr_{n+1}^{e}$ is generated, by using a subset of valid crime reports $CR_{e}^{v}\subseteq CR_{e}$ such that $CR_{e}^{v}\neq \emptyset$. That means, every time a new crime report $cr_{n+j}^{e}$ (with j≥1) is generated, it is added to the $CR_{e}$ set; however, only a subset of them $CR_{e}^{v}$ are used to calculate the new associated tracking point $tp_{n+j}^{e}$.

In order to determine which crime report is valid and, as a consequence, part of the $CR_{e}^{v}$ set, a *time window-based* approach is adopted. More specifically, given a *Time Window* TW, and let us suppose that a new crime report $cr_{n+1}^{e}\left(ts\right)$ is generated at time ts, then the $CR_{e}^{v}\left(ts\right)$, calculated at time ts, includes the last crime report $cr_{n+1}^{e}\left(ts\right)$, as well as all the crime reports $cr_{i}$ that were generated in a period of time δt such that $cr_{n+1}^{e}\left(ts\right)-cr_{i}^{e}\left(t\right)=\delta t<TW$, as defined in Equation (2).

(2) $$CR_{e}^{v}\left(ts\right)=cr_{n+1}^{e}\left(ts\right)\cup \left\{cr_{i}^{e}\left(t\right)\in CR_{e}\right\}⇐ts-t<TW,\;\forall i=1,\ldots ,n$$

As a consequence, on the basis of the available valid crime reports $CR_{e}^{v}$, two cases are possible for computing a new tracking point $tp^{e}\left(t\right)$, as expressed through the Equation (3):

- *case 1*: when only one crime report $cr^{e}$ is valid, that is, $|CR_{e}^{v}|=1$, then the user coordinates $uc_{k}$, with k={longitude,latitude}, associated to the $cr^{e}$, coincide to the coordinates of the tracking point $tp^{e}$, and the timestamps ts of the crime report $cr^{e}\left(ts\right)$ is the timestamp of the generated tracking point $tp^{e}\left(t\right)$, that is t=ts.
- *case 2*: when at least two crime reports are valid, that is, $|CR_{e}^{v}|\geq 2$, then the coordinates used to generate the coordinates of the tracking point tp are calculated as the mean value of the coordinate component $uc_{k}$, with k={longitude,latitude}, associated to each crime report $cr^{e}\in CR_{e}^{v}$, whereas the timestamp *t* associated to the new tracking point $tp^{e}\left(t\right)$ coincides to the most recent ts associated to the last crime report $cr^{e}\left(ts\right)$.

(3) $$tp^{e}\left(t\right)=\left\{k^{e}\left(t\right)\right\}\left\{\begin{array}{c} \;uc_{k}^{cr^{e}\left(ts\right)}\;with\;t=ts⇐cr^{e}\in CR_{e}^{v}\;and\;\left|CR_{e}^{v}\right|=1,\forall k=\left\{longitude,latitude\right\}\\ \;\frac{\sum_{}^{}uc_{k}^{cr^{e}\left(ts\right)}}{|CR_{e}^{v}|}\;with\;t=max\left(ts\right)⇐\left|CR_{e}^{v}\right|\geq 2,\;cr^{e}\in CR_{e}^{v},\;\forall k=\left\{longitude,latitude\right\} \end{array}\right.$$

**Figure 3:**
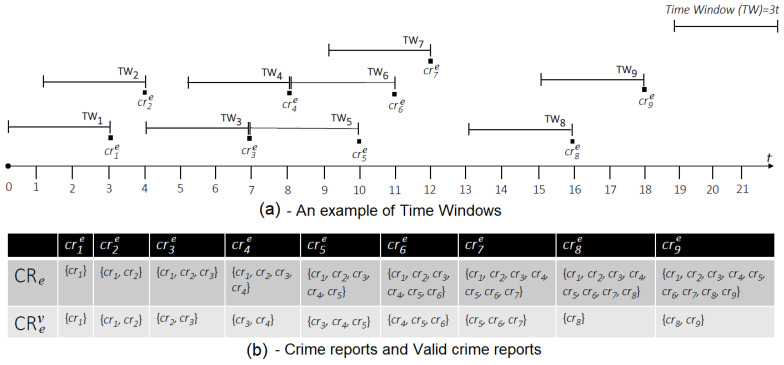
Working mechanims for the selection of valid crime reports based on the time window approach.

Figure 3 exemplifies the working mechanism for selecting a valid set of crime reports, in order to generate new tracking points, by using a *Time Window* with *TW = 3* time units. Specifically, the diagram of Figure 3a shows the temporal sequence of the crime reports $cr_{1}^{e},cr_{2}^{e},\ldots ,cr_{9}^{e}$, along with the graphic representation of the time windows associated to each individual crime report. The table in Figure 3b, instead, illustrates for each crime report all history of crime reports $CR_{e}$ so far generated, as well as only the subset $CR_{e}^{v}$ of valid crime reports related to the event *e*, everytime a new crime report $cr_{i}^{e}$, with i=1,…,9, is generated.

Algorithm 2 reports the pseudocode that has been defined on the top of the above-presented concepts, which in turn is formalized through Equations (2) and (3). In particular, given as input a crime report cr and a crime event *e*, then a tracking point $tp^{e}$ is calculated. Specifically, if *e* is a new event and it does not have any cr associated to it, then a new set of crime event $CR_{e}$, which coincides with the $CR_{e}^{v}$, is initialized with the first crime report cr, otherwise it is added to the existing set, and the $CR_{e}^{v}$ is updated on the basis of the time window TW = δt. The $tp^{e}$ is then computed on basis of the updated $CR_{e}^{v}$ version.

**Algorithm 2:** Computation of a Tracking Point

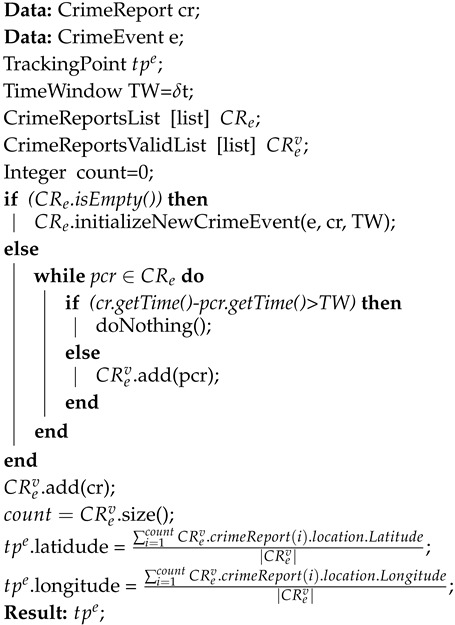



As in an arbitrary period of time different crime events *e* can take place, several crime reports cr could be generated. However, it does not mean that all crime reports are linked to the same crime event and that they belong to the same tracking point set. As a consequence, it is necessary to establish which crime report refers to which crime event, that in turn is used to compute its related tracking points.

To deal with this issue, another aspect regarding the spatial occurrence of a crime, which in turn is related to the distribution of the citizens, has been considered. In particular, based on the idea that two or more entities (in this case the “citizens”) are considered related to each other if they are close (i.e., if they are in the same area within a time window), a *proximity principle* has been adopted. On the basis of such principle, the *Range of Action (RoA)* of a crime event *e* has been defined as the physical area (i.e., the maximal distance) within the crime *e* can be perceived during the “Crime Occurrence” or “Crime Evolution” phase.

Specifically, given a crime report $cr_{i}\left(t+\Delta t\right)$ and an arbitrary crime event *e* tracked through its latest tracking point $tp^{e}\left(t\right)$, their distance is defined as the distance between their locations *dist($cr_{i}$, $tp^{e}$) = $|cr_{location}^{i}$ − $tp_{location}^{e}|$*. If *dist($cr_{i}$, $tp^{e}$) < RoA*, then the crime report is $cr_{i}\left(t+\Delta t\right)$ is considered close to the crime event *e*. Given a set of crime event $E=\{e_{1},e_{2},\ldots ,e_{j},\ldots ,e_{m}\}$, then the crime report $cr_{i}$ is associated to the event $e_{j}$ with the minimum distance from it; otherwise, a new event $e_{m+1}$ is initialized and associated to $cr_{i}$ according to Equation (4).

(4) $$cr_{i}^{e}\left(t+\Delta t\right)=\left\{\begin{array}{c} cr_{i}^{e_{j}}\left(t+\Delta t\right)⇐\exists \;e_{j}\in E\;s.t.\;Min\left(dist\left(cr_{i},tp^{e_{j}}\right)<RoA\right),E=\left\{e_{1},e_{2},\ldots ,e_{j},\ldots ,e_{m}\right\}\\ cr_{i}^{e_{m+1}}\left(t+\Delta t\right),\neg \exists \;e_{j}\in E\;s.t.\;e_{m+1}=e_{j},\;E=\left\{e_{1},e_{2},\ldots ,e_{j},\ldots ,e_{m}\right\} \end{array}\right.$$

The pseudocode, which implements the allocation mechanisms of a crime report to a crime event based on the proximity principle above-presented, is reported through Algorithm 3.

**Algorithm 3:** Proximity principle-based allocation of a crime report cr to an event *e*

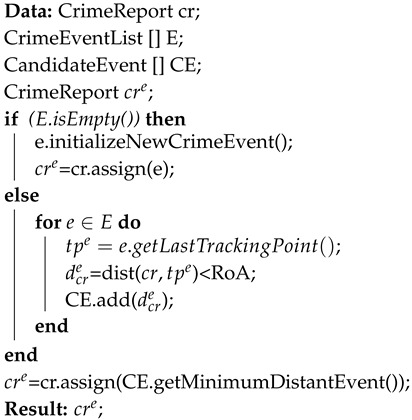



**Figure 4:**
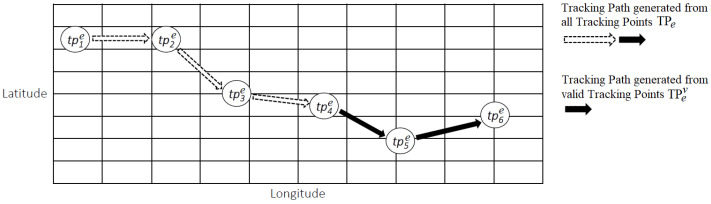
An example of a tracking path computation based on tracking points.

The above-defined concepts are employed to group different crime reports cr, whose information is used to generate *Tracking Points $tp^{e}$* related to the same crime event *e*, in order to track it over the time. On the basis of the crime reporting set related to a specific event of crime $CR_{e}$ a tracking path related to a crime event *e* can be generated $TP_{e}=\left\{tp_{1}^{e},tp_{2}^{e},\ldots ,tp_{j-1}^{e},tp_{j}^{e}\right\}$, which is defined as the spatio-temporal sequence of tracking points $tp_{i}^{e}\in TP_{e}$ associated to a specific crime event *e*, as it is depicted in Figure 4.

### 4.4. Path Prediction and Back Notification

The path prediction and back notification concepts aim to tackle the third and fourth questions discussed in Section 3, in order to predict the moves of a criminal and to what extent, as well as to improve the safety of the citizens.

As presented in the previous subsection, the history of a criminal’s movement relies on the sequence of tracking points $TP_{e}$, which in turn can be used to derive further information, in order to make prediction, for example, by analyzing its direction. However, during the evolution of the crime, the logic of the criminal movement and, as a consequence, of the data generated accordingly, could change due to unexpected conditions, such as a police checkpoint, or a wall that does not allow to keep on going straight ahead, and so on. Such conditions entail a change in the movements, and therefore the logic of the new route may be inconsistent with the logic of the previous stretch. Consequently, a mechanism to flexibly re-adapt and re-interpret the criminal’s moves is in needed. In this context, the logic adopted to select the valid crime reporting set has been similarly adopted to deal with the prediction of a criminals’ movements, by using only the most recent tracking points, which are called valid tracking points.

In particular, a valid tracking point set $TP_{e}^{v}\subseteq TP_{e}$ is defined as a subset of tracking points which are considered valid. Given a crime event *e*, a set of tracking points $TP_{e}$ related to *e*, an arbitrary Time Window TW, and a tracking point $tp_{j}^{e}\left(t_{j}\right)$ generated at time $t_{j}$, then a valid tracking point set $TP_{e}^{v}$ can be computed in two ways, according to Equation (5).

(5) $$TP_{e}^{v}=\left\{\begin{array}{c} \left\{tp_{j}^{e}\left(t_{j}\right)\right\}\equiv \left\{tp_{1}^{e}\left(t_{1}\right)\right\}⇐\left|TP_{e}\right|=\emptyset ,that\;is\;∄tp_{i}^{e}\left(t_{i}\right)\in TP_{e}\;such\;that\;tp_{i}^{e}\left(t_{i}\right)\neq tp_{j}^{e}\left(t_{j}\right)\;and\;t_{i}<t_{j},\\ \left\{tp_{j}^{e}\left(t_{j}\right),tp_{j-1}^{e}\left(t_{j-1}\right)\right\}\cup tp_{i}^{e}\left(t_{i}\right)\in TP_{e}s.t.t_{j}-t_{i}<TW,\forall i=\left\{1,2,\ldots ,j-2\right\}⇐\left|TP_{e}\right|\neq \emptyset . \end{array}\right.$$

The first case corresponds to the generation of the first tracking point $tp_{j}^{e}\left(t_{j}\right)\equiv tp_{1}^{e}\left(t_{1}\right)$ associated to the crime event *e*. Consequently, the valid tracking point set $TP_{e}^{v}$ has one valid tracking point, and does not exist for any other tracking point associated with this crime event $tp_{i}^{e}\left(t_{i}\right)\in TP_{e}$, whose generation time $t_{i}$ is earlier, $t_{i}<t_{j}$, whereas the second case takes place when a tracking point $tp_{j}^{e}\left(t_{j}\right)$ associated to a crime event *e* is generated and other tracking points exist in the tracking point set $tp_{i}^{e}\left(t_{i}\right)\in TP_{e}$. In this case, the valid tracking point set $TP_{e}^{v}$ basically consists of the latest generated tracking point $tp_{j}^{e}\left(t_{j}\right)$ and the penultimate $tp_{j-1}^{e}\left(t_{j-1}\right)$, as well as other tracking points $tp_{i}^{e}\left(t_{i}\right)$ if and only if their generation time $t_{i}$ falls within the time window TW, that is, $t_{j}-t_{i}<TW$.

**Figure 5:**
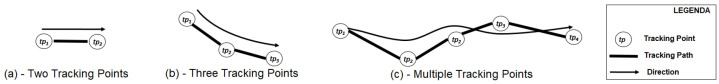
Direction.

**Figure 6:**
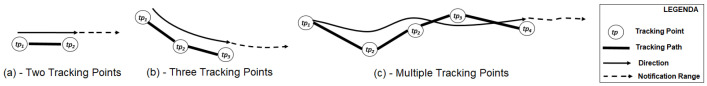
Direction-Prediction.

**Figure 7:**
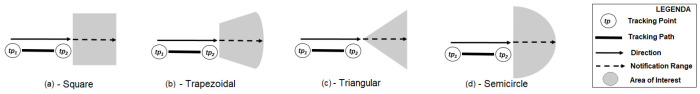
Direction-Prediction-Area Of Risk.

As depicted in Figure 4, by using the valid tracking points set $TP_{e}^{v}=\left\{tp_{4}^{e},tp_{5}^{e},p_{6}^{e}\right\}$, a valid tracking path, which is represented through the black arrows, is generated. On the basis of the valid tracking path, the *Path Prediction* and *Back Notification* mechanisms are defined. In particular, (i) Path Prediction, in order to predict the potential movements of a criminal, and (ii) Back Notification, in order to decide who has to be notified about the current or upcoming danger, by considering the following three parameters; the *direction*, the *notification range* (or *prediction range*), and the *area of risk*. More specifically:

- *direction*: given two or more tracking points, this parameter characterizes the movement towards which a criminal is potentially going, as it is represented in Figure 5, and consequently to predict where the crime is evolving in near future.
- *prediction/notification range*: given an arbitrary direction, this parameter refers to the distance within which the prediction must be made (e.g., 10 m, 100 m, and 1000 m), as it is shown in Figure 6. The greater the distance the more hypotheses can be conducted; however, an excessive distance could lead to unreliable predictions due to unforeseen situations, as already above-discussed.
- *area of risk*: on the basis of the given direction and the prediction range, this parameter allows to circumscribes the area, according to different shapes, as depicted in Figure 7, within the prediction will be performed as well as to select to whom notifications have to be sent.

Once the area of risk if identified, this information can be strategically used from the police officers. For example, knowing what is around that area might help the police officers to figure out why a criminal is moving there, and then to anticipate the next move. For instance, if in the area there is a train station, that might mean that the criminal is trying to escape. Such information can be used to notify local security around it, as well as to send some police guards over there in order to catch the criminal. Whereas, if it is a residential area with a lot of restaurants or shopping centers, it might mean that the criminal might be dangerous for the people, at this point the system can be used to send back notifications to the users in this area by alerting them or by suggesting potential actions in order to make them safer.

Algorithm 4 reports the pseudocode of the above presented concepts for supporting the identification of the direction, area of risk as well as back notification. Its current implementation is based on the use of two tracking points for the identification of the direction, (as it is represented in Figure 5a), the approach depicted in Figure 6a for the notification range, whereas it approximates the area of risk by using a triangular area (see Figure 7c). Every time a new tracking point $tp^{e}$, related to a specific event *e*, is generated, a new direction and the related area of risk is updated.

**Algorithm 4:** Prediction and User Notification

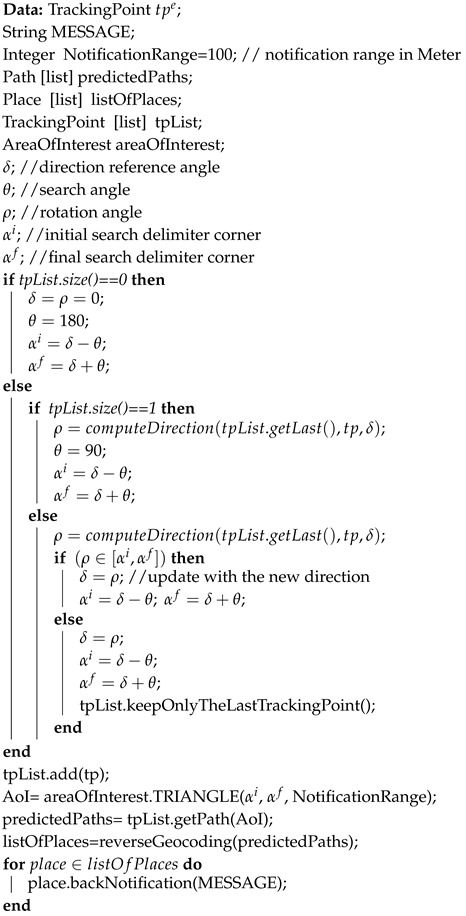



Once the area of risk is identified, all possible streets within the area are collected and then, by using reverse geocoding, both (i) the places located around the streets are extracted and (ii) citizens in that area are identified. From one side the identified places are used and analyzed by the police in order to understand where the criminal could want to go. This supports his interception in terms of anticipating his moves, by sending police officers on the spot in advance. From the other side, the users present in the current area of risk can be reached online and supported via an information service based on targeted safety back notification messages.

## 5. System Implementation

In this section, the technical description of the system is presented. It is centered on the architectural model as well as on the implementation of the concepts and algorithms, elaborated in the previous section.

**Figure 8:**
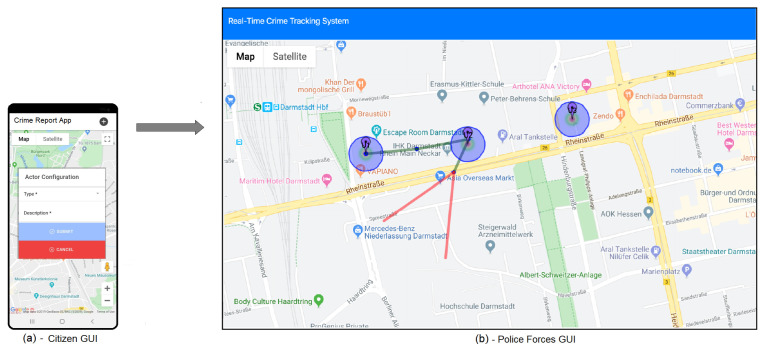
Tracing criminal events: a tool overview in Operation Mode.

A general overview of the system is depicted in Figure 8. In particular, Figure 8a shows the Graphical User Interface (GUI) from the citizens/users side, which implements the IoT Layer of the logical architecture described in Section 4.1 as a mobile App. It allows citizens to generated *crime reports* according to the concepts defined in Section 4.2. It worth noticing that, when generating a crime report, *location* and *timestamp* are automatically extracted from the mobile device, whereas *type of crime* and *description* can be additionally inserted by the user. Figure 8b shows the GUI from the Police Forces perspective. It implements the other layers of the logical architecture described in Section 4.1, according to the concepts defined respectively in Section 4.3 and Section 4.4. In particular, a blu circle on the map represents a citizen, who can interact with the system by generating *crime reports* and receiving *back notifications* through the app, whereas the calculated *tracking path* is represented as green segments interconnected by *tracking points* depicted as smaller circles. The *predicted direction* and the *area of risk* is, instead, delineated from the triangular area enclosed between the two red segments.

This configuration represents the so called *operation mode*. It illustrates how the system works in reality, in which an App enables the citizens both to send information to the PFs and to receive notification from them, when a crime event occurs. In order to show its functioning and to test the algorithms, instead, a simulation-based approach has been adopted. As a consequence, another functioning mode, called *simulation mode*, which is described in following subsection, has been introduced.

### 5.1. Simulation-Based Approach

The *simulation mode* extends the *operation mode* by enabling the simulation of a criminal scenario by introducing two types of virtual actors: a *VirtualCitizen* and a *VirtualCriminal*. Such virtual actors replace the real users, in the generation process of crime reports, centered on the mobile app, when perceiving a real criminal, by implementing a simulated perception. More specifically, a *VirtualCriminal* is used to simulate the behavior of a criminal by generating a crime event in the scenario, whereas a *VirtualCitizen* simulates the behavior of a citizen, who can send and receive notifications about an event of crime.

**Figure 9:**
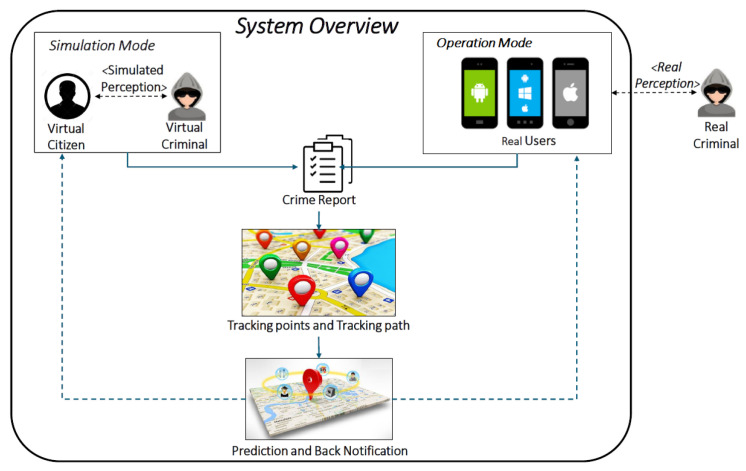
Dual mode system functioning.

As illustrated in Figure 9, in the *operation mode* (i.e., in reality) the generation of a crime report from a user is guided by the physical perception of a crime event. For example, at the sight of the criminal who is committing a crime, or being part/victim of a crime situation. In order to simulate this mechanism, in the *simulation mode*, not only the role of the virtual citizen has been implemented, but also the role of the virtual criminal has been introduced in the simulator. This is necessary to simulate the perception of a crime situation, in order to trigger the virtual citizen to generate a crime report. As a consequence, when the software is working in *simulation mode*, it includes all the architectural layers, that are described in Section 4.1.

**Figure 10:**
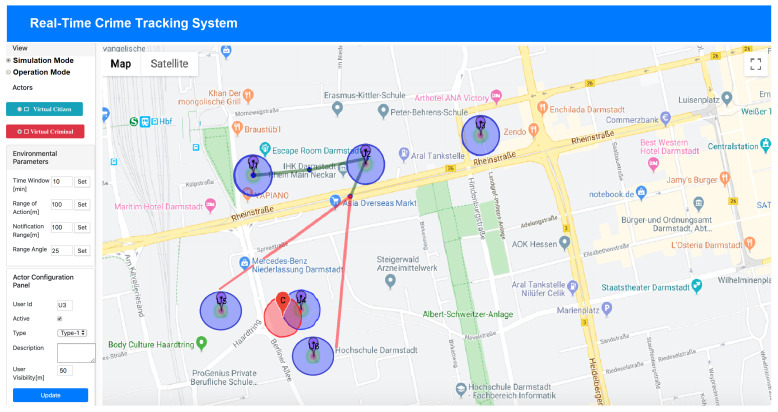
Tracking criminal events: an overview of the software environment.

Figure 10 shows the GUI of the system in simulation mode. It is built on the top of Google maps, as it allows natively to visualize graphically streets and places. More details of the overall employed technologies for its implementation is provided below.

In the center of Figure 10 is the map, which differs from that of Figure 8b as the blue circles represent *VirtualCitizens*, while the red circle models the concept of the *Virtual Criminal*. On the top-left part of the software environment, it is possible to select the functioning mode: *Operation mode* or *Simulation mode* (as it is currently depicted). Below it, a panel for selecting *VirtualCitizen(s)* and *VirtualCriminal(s)* is available. The *VirtualCriminal*, who plays the role of criminal, is used to simulate the behavior of a person who commits a crime and, as a consequence, it is used to trigger the event for the *VirtualCitizens* in order to generate crime reports. More specifically, when the simulation mode is active, it is possible to select, through the respective buttons, virtual citizens and virtual criminals to be placed on the map and to configure them, in order to set up a simulated criminal scenario.

In particular, the *Actor Configuration Panel* is available on the left bottom side of the GUI which allows to configure *VirtualCitizen*. Through it, it is possible to configure each of such actors, in such a way that they are able to warn dangerous events (if the option “Active” is checked) when they perceive a close *Virtual Criminals*, which is specified in meter through the parameter “UserVisibility”.

The concept of perception is modeled using the size of the circle that is used to represent virtual actors, whose radius can be defined through the *UserVisibility* parameter. In particular, a *Virtual Citizen* perceives a criminal or a crime, when the circle representing him intersects the circle of a *Virtual Criminal*. At this point the *Virtual Citizen* is triggered to generate a report. As a consequence, the bigger a circle is configured the bigger is the possibility of a *Virtual Citizen* to get in touch with a *Virtual Criminal* through the intersection. The report is generated according to the Algorithm (1) by additionally specifying the “Type of crime” and an optional “ Textual Description”). Virtual actors with such configuration are used to simulate the citizens who, in the real life, have the App installed and active on their smart devices.

On the left middle side of the GUI, a configuration panel called *Environmental Parameters* allows, instead, to configure the parameters which are used to compute both the *Tracking points* and the *Area of risk*. In particular:

- the *Time Window* is computed in minutes, and it is used both to select the most recent crime reports for generating tracking points, according to the concept defined in Section 4.3 and Section 4.4;
- the *Range of Action* is computed in meters, and it is used to allocate crime reports to events of crime, on the basis of the proximity principle defined in Section 4.3;
- the *Notification Range* is defined in meters, and it is used to determine area of risk about the ongoing potential dangerous situation as well as whom to send the notifications on the basis of the concepts defined in Section 4.4;
- the *Range Angle*, which is computed in angular degree, is used to define the size of the area of risk in order to delimit the places to be gathered as well as the citizens to be notified.

Before discussing the data gathered by running the tool in simulation mode, in order to illustrate how the defined algorithms work, a brief overview of the digital technologies, which have been employed to implement the above-presented system, is provided.

In particular, *JavaScript* [47] is a lightweight scripting language for developing web pages that allows to write client-side code, by making dynamic pages, in order to support the interaction with users. The main advantages rely on being client-side, that means to be very fast because it can be run immediately within the client-side browser, interoperable with other programming languages, and extendable in terms of functionalities. Whereas, *Node.js* [48] is a runtime environment for the server-side scripting execution. It is open source and cross-platform and it supports the generation of dynamic web page content before the page is sent to the user’s web browser [49]. It is built on the V8 Chrome engine, and it is event-based and non-blocking. It means that it is able to have multiple requests in progress at the same time by the same process (or thread), as it uses a non-blocking I/O model. *Firebase* [50] is a Backend-as-a-Service (BaaS) app development platform, which supports data storage and sending notifications [51]. Whereas, Firebase Cloud Messaging (FCM) is a free cross-platform solution for messages and notifications for Android, iOS, and web applications, which provides a real-time database and backend as a service. APIs, for the developers, are also available [52]. *Google Maps* [53] is a web mapping service, which provides APIs to build location-based services, that supports JavaScript. It offers satellite imagery; aerial photography; street maps; 360 panoramic views of streets (Street View); real-time traffic conditions; and route planning for traveling by foot, car, bicycle, air, or public transportation. *Ionic Framework* [54] is a mobile UI toolkit which provides a free and open source SDK for developing cross-platform mobile application for native iOS, Android, from a single codebase [55]. Whereas, *AJAX* [56] is a client side technique for communication with a web server. It focusses on the exchanging data with a server, and update parts of a web page without reloading the whole page, by decoupling the data interchange layer from the presentation layer [57].

### 5.2. Evaluation and Discussion

The evaluation was based on three different approaches: (i) a simulation-based experimentation to assess the functioning on the defined algorithms, (ii) an usability-based evaluation in order to assess the system in terms of its usability from the citizens perspective, and (iii) a statistics based approach in order to estimate the intervention time gained from the PFs by using it.

The experimentation of the above presented implementation has been tested on a city scale, and in particular in Darmstadt (Germany). Figure 10 represents the area close to the train station of the simulated scenario, which has been used to test the proposed concepts and the related algorithms. It shows four *Virtual Citizens*, that have been used and configured as “Active”, and one *Virtual Criminal*. The simulation is performed by manually moving the *Virtual Criminal* on the map. When the *Virtual Criminal* enters in the range of visibility of a *Virtual Citizen* (i.e., close to a citizen according its “UserVisibility”), a crime report is automatically generated by such specific *Virtual Citizen*. The data coming from each report consists of the timestamp, the citizen coordinates, and eventually the type of crime and additional non-structured details, in terms of textual description. Table 4 reports the temporal sequence of the data related to the crime reports generation.

In particular, the data in column “Crime Id” keeps on tracking who generated the crime report, whereas the generation time of the report is described in column “Report Timestamp”. In the column “User Coordinates” the location of the report are expressed through latitude and longitude. Additional data are provided through the other columns such as “Crime Type” and “Textual Description”.

It is worth noting that, as it is stated in Section 4.2, both “Crime Type” and “Textual Description” are optional data. As a consequence, they are not used in the automatic computation process for the detection and tracking of criminals. However, if they are provided by the reporters, they can be afterwards analyzed from the LEAs to make off-line decisions, or elaborating statistic, such as the most popular crime type within a specific area, or in which period of the year a certain type of crime occurs most, and so on.

The crime reports generated from the citizens are used for the computation of the tracking points. As explained before, every time a new crime report is received by the system, a new tracking point is generated. Table 5 shows the computation of three tracking points according to the temporal receivement of each crime report of Table 4. In particular, in the column “Tracking Point Id”, the identifier of a tracking point is reported, whereas the column “Id Valid Crime Report” shows the valid crime report set $CR_{e}^{v}$ usd to to compute the tracking point under consideration. The columns “Generated Location” and “Reference Time” report the spatial and temporal value assigned to the calculated tracking point, as explained in Section 4.3.

For example, the first Tracking Point with Id = “@01” coincides exactly with the information coming from the first report, that is, the Generated Location are the Users Coordinates and the Reference Time is the Report Timestamp. The Tracking Point with Id = “@02” is computed when the second crime notification is generated. In this case, this tracking point is computed by exploiting the data of the last notification and the previous one, because both of them have been generated within the *Time Window* that is set equal to “10 min”. The Reference Time is always the Report Timestamp of the last crime report, whereas the Generated Location is calculated as the mean value among both the User Coordinates related to their crime reports. The Tracking Point with Id = “@03” is generated when the third Crime Report is received. As it is shown in Table 5, the Tracking Point with Id = “@03 ” is computed only using the Crime Report with Id = 02 and Id = 04, as stated in the “Id Valid Crime Report” list. This is because the data gathered from the Crime Report with Id = 01 is considered already old, as it does not fall anymore within the Time Window. Consequently, it is considered obsolete/not valid, and then discarded for the computation of the next tracking point.

Table 6, instead, provides an extract of the Predicted Places where the information has to be sent in order to inform people about a potential threatening event coming. In particular, the prediction with Id = “#01” does not provide any important information as it is based on one single Tracking Point. Whereas both the prediction with Id = “#02” and Id = “#03”, as they are based on at least two Tracking Points, provide indications about the possible directions and, as a consequence, places towards the criminal is moving. For example, the indication gathered from the prediction with Id = “#03” is graphically shown in Figure 10, where the tool is highlighting in red color the so called area or risk. Such information can be used both (i) to alert people around specific areas of the city about the potential threat and specifically the “Users in the Area of Risk”, and (ii) to improve the moves of the Police Forces by knowing in advance potential places and locations, called in the table “Place Information”, where a criminal might go.

Further evaluation has been conducted by assessing the real system in terms of user usability. We considered it is important to know people’s opinion and receive their feedback, as the ease of use of the crime notification system, in moments of panic, is of fundamental importance, to guarantee the effectiveness and the willingness to use this tool. This is the reason to keep it as simple as possible for making its use efficient and fast from the user perspective. To this aim, an user study has been conducted, by systematically examining the characteristics and behavior of the systems and services. The user study has been directly linked with the effectiveness (performance) and information services provided, as they aim at satisfaction of user needs.

In particular, the user study was carried out for the mobile application, by yielding qualitative indications on the goodness of the proposed system. The survey was conducted on a sample of 30 people between 18 and 50 years of both genders, by evaluating the following aspects: (i) *Content and Navigation*—the quality of being able to find content in a simple and intuitive way; (ii) *Functionality*—the quality of being suited to serve a purpose well; (iii) *Look and Feel*—the quality of the graphical user interface and aspects of its design, including elements such as colors, shapes, layout, and typefaces, as well as the behavior of dynamic elements such as buttons, boxes, and menus; (iv) *Response Time*: the total time that the system takes to react to a given input, such as loading data as well as sending a request; (v) *Stability*—which is a measure of the repeatability of a test over time, that gives the same results whenever it is used.

**Figure 11:**
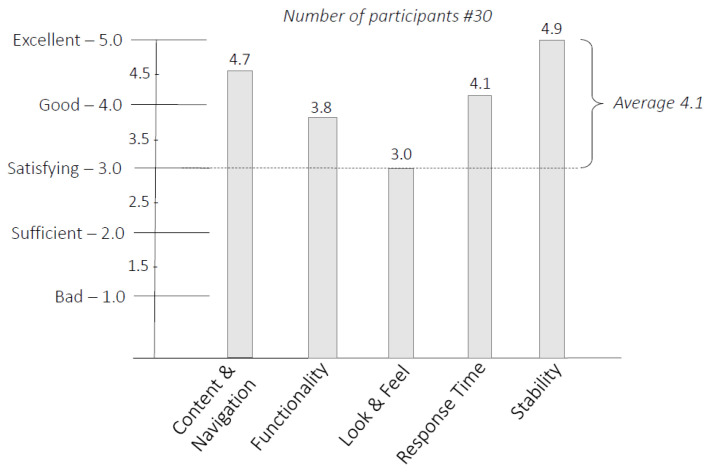
Results of the qualitative analysis based on the user study.

Figure 11 shows the mean value for each of those aspects (i.e., 4.7, 3.8, 3.0, 4.1, and 4.9), by highlighting a qualitative assessment, more than positive. In fact, not only the lowest mean value is greater than 3.0 (i.e., Satisfactory), but the average of all values is 4.1, which means better than Good.

Furthermore, based on the availability of statistical results (i.e., location detection time of the crime and intervention time) [58,59] related to four well-known cities (i.e., San Francisco, Houston, Los Angeles, and New York) with a crime rate between 50 and 65 according to [60], the advantage in terms of intervention time, gained from the PFs by using the App, has been estimated. Whereas Darmstadt was not included in this estimate as it has a low crime rate, equal to 26.05, and data regarding the crime location detection time and response time is not available.

**Figure 12:**
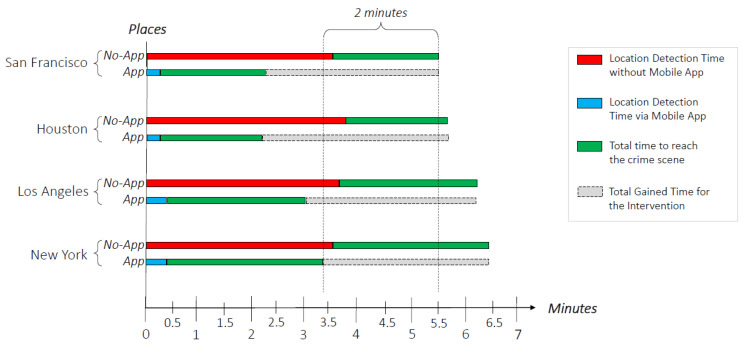
Location detection and gained intervention time estimation.

As it is represented in Figure 12, the overall response time of the PFs is in average more than 5 min. It depends from the total time to reach the crime scene (depicted in green color), and it is strongly impacted from the Location Detection Time (depicted in red color), which takes at least 3.5 min to be identified. By using the location received through the app (depicted in blue color) the information about the crime scene is automatically gathered on the basis on the speed of the network and, as a consequence, it is detected in few seconds. Consequently, the overall response time decreases, as it mainly depends only from the time to physically reach the crime scene.

As it is represented in Figure 12, from the analysis conducted by using the data of the above cities, for each city the intervention time would be reduced by about 3 min (as it is highlighted in gray color). In addition, by considering the worst case, that is given by the difference between the overall Response Time in New York using the App and the Total Response Time in San Francisco without App, it is more than 2 min, which might have a significant impact on real life in terms of human safety.

## 6. Conclusions

The paper focused on the tracking of crime events and criminals by involving the citizens in the crime detection loop of the Police Forces (PFs) by relying on IoT devices and an edge computing approach.

In particular, (i) a system based on IoT devices, for enabling the citizens to actively participate in the crime detection process, has been proposed; (ii) a mechanism for tracking the evolution of crimes and of criminals has been defined; (iii) an approach for the movement prediction regarding potential directions of criminals, has been elaborated along with (iv) a back notification mechanism, which enables the LEAs to communicate back to the users, was implemented. The concepts of *Crime Report, Tracking point, Tracking path, Prediction* and *Back Notification*, along with specific algorithms to track a crime and enable the communication among citizens and LEAs have been elaborated.

Finally, an implementation, based on IoT social devices and centered on mobile Apps has been realized. It relies on a edge-based architecture that is logically structured as a 4-layer system. The *IoT layer* enables the citizens both to communicate the occurrence of criminal events as well as to receive *Back Notification* from the PFs. The *Edge layer* represents the layer in which the raw data, coming from different devices, is collected and pre-elaborated. In the *Analysis layer*, the pre-elaborated data is used by the local PFs for (i) tracking crime events and criminals (ii) understanding towards a criminal is potentially going (iii) alerting citizens by sending them back notifications. The *Global layer* allows the national PFs to further elaborate information in order to support off-line decisions, for example at national level.

The logic of the system has been assessed through simulation techniques that allowed not only to test the algorithms and the functionalities in a virtual environment, but it also enabled (i) the setting of the parameters during its design, (ii) the identification of abnormal or unexpected behavior, and (iii) to refine the concepts and the algorithms before its deployment. Furthermore, the usability of the system, from the citizens perspective, was assessed through a survey where 30 participants have rated 5 aspects. From the survey emerged that all the evaluated aspects reached at least the value of 3.0 (Satisfying) and an average of 4.1 (i.e., between Good and Excellent) has been achieved. Finally, a statistical approach was adopted to evaluate the time employed from the PFs to reach the crime scene, after receiving the information from the citizens by phone calls in comparison with the information received via App. This analysis showed that in average at least 3 min can be saved, and by comparing the worst and best cases, 2 min are still gained. Future works might include a further analysis by comparing the estimated evaluation with the response of the PF coming from the real usage of the propose system in reality.

## Figures and Tables

**Table 1:** A summary of the related works on crime and emergency situations.

Related	Device Type	Used	Main	Evaluation
Work	& Approach	Feature	Goal	Method
[22]	Smartphone,	Location,	Risky areas information,	User study
	Client-Server	Timestamp	Closest police station	
[23]	Smartphone	Location,	Crime distribution	Not-stated
	Client-Server	Image, Audio	information	
[24]	Smartphone	Location, Phone Number,	Emergency units	Not-stated
	Client-Server	User Name, Age	dispatching	
[25]	Smartphone	Location, Text, Audio,	Emergency	Usability Test,
	Client-Server	Video, Image, Time	reporting	Simulated Emergency
[26]	Smartphone	Location, Emergency Type,	Emergency	User study
	Client-Server	Sensitive data	reporting	
[27]	Smartphone	Location,	Crime	User study
	Client-Server	Text, Video	reporting	
[4]	Wearable	Heartrate,	Crime detection,	Not-stated
	Client-Server	Body Temperature	Crime prediction	

**Table 2:** A comparison of most popular apps related to emergency situations.

Related	App	App	Real-Time	Mobile	Path
Work	Name	Category	Reporting	Notification	Prediction
[28]	Red Panic Button	Short-Cut	Yes	No	No
[29]	SafeTrek	Short-Cut	No	Yes	No
[30]	bSafe	Short-Cut	Yes	No	No
[37]	Safety Guardian	Geo-Fancing	Yes	Yes	No
[38]	Watch Over Me	Geo-Fancing	Yes	Yes	No
[39]	LifeLine Response	Geo-Fancing	Yes	Yes	No
[33]	Citizen	Crime/Disaster Tracking	No	Yes	No
[35]	Crime Mapping	Crime/Disaster Tracking	No	No	No
[36]	Disaster Alert	Crime/Disaster Tracking	No	Yes	No
[34]	Warn-App NINA	Crime/Disaster Tracking	No	Yes	No
[27]	Ustream	Crime/Disaster Tracking	Yes	No	No

**Table 3:** Identified features and related description.

Feature	Description
User Coordinates (uc)	They are used to characterize the user’s position near the crime scene, GPS-based
Report Timestamp (ts)	It is used to establish the temporal occurrence of a crime event,
	that is, when it is perceived as a threat
Crime Type (ct)	It is a data that a user can report in order to provide an information about the type
	of threats (e.g., anti-social behavior, violent crime, criminal damage and arson,
	shooting, burglary, shoplifting, vehicle crime, public order, bicycle theft, other theft,
	drugs, theft from the person, robbery, possession of weapons, other crime)
Textual Description (td)	It is an optional description that could be provided from the user
	to further describe and better characterize the ongoing event

**Table 4:** Data related to different crime reports.

Crime	Report	User	Crime	Textual
Id	Timestamp	Coordinates	Type	Description
01	20190215-154022	50.105024, 8.647333	robbery	-
02	20190215-154935	50.106380, 8.650863	weapon	-
04	20190215-155802	50.106903, 8.654651	other	-
...	...	...	...	...

**Table 5:** Tracking point computation.

Tracking	Id Valid	Generated	Reference
Point Id	Crime Report	Location	Time
@01	{01}	50.105024, 8.647333	20190215-154022
@02	{01, 02}	50.104983, 8.648020	20190215-154935
@03	{02, 04}	50.106398, 8.653040	20190215-155802
...	...	...	...

**Table 6:** Prediction and User back notification.

Id	Tracking	Predicted	Place	Users in the
Prediction	Point List	Places	Information	Area of Risk
#01	{@01}	-	-	-
#02	{@01, @02}	50.109631, 8.652664	Al Rahma Mosque	{U03}
#03	{@02, @03}	50.107073, 8.663131,	Train Station,	{U05, U06}
		50.110071, 8.663655	Mercedes-Benz	
...	...	...	...	...

## Abbreviations

The following abbreviations are used in this manuscript:

LEAs	Law Enforcement Agencies
PFs	Police Forces
LPFs	Local Police Forces
NPFs	National Police Forces
ESs	Emergency Services
IoTs	Internet of Things
CS	Collaborative System
MCS	Mobile Collaborative System
CRS	Criminal Record System
SVS	Social Video Streaming
GPS	Global Position System
FCM	Firebase Could Messaging
CT	Crime Type
UC(s)	User Coordinate(s)
TS	Report Timestamp
TD	Textual Description
CR	Crime Report
TP	Tracking Point
TW	Time Window
RoA	Range of Action
GUI	Graphical User Interface
TPA	Tracking Path
PP	Path Prediction
BN	Back Notification

## References

[1] Magen A. (2018). Fighting Terrorism: The Democracy Advantage. J. Democr..

[2] Tundis A., Kaleem H., Muhlhauser M. Tracking Criminal Events through IoT Devices and an Edge Computing Approach. Proceedings of the 2019 28th International Conference on Computer Communication and Networks (ICCCN).

[3] (2019). EU H2020 TAKEDOWN Research Project. https://www.takedownproject.eu/.

[4] Byun J.-Y., Nasridinov A., Park Y.-H. (2014). Internet of things for smart crime detection. Contemp. Eng. Sci..

[5] Tundis A., Bhatia G., Jain A., Muhlhauser M. Supporting the Identification and the Assessment of Suspicious Users on Twitter Social Media. Proceedings of the 2018 IEEE 17th International Symposium on Network Computing and Applications (NCA).

[6] Tundis A., Jain A., Bhatia G., Muhlhauser M. Similarity Analysis of Criminals on Social Networks: An Example on Twitter. Proceedings of the 2019 28th International Conference on Computer Communication and Networks (ICCCN).

[7] Tundis A., Ruppert S., Mühlhäuser M. (2020). On the Automated Assessment of Open-Source Cyber Threat Intelligence Sources. International Conference on Computational Science.

[8] Number of Mobile Phone Users Worldwide from 2015 to 2020. https://www.statista.com/statistics/274774/forecast-of-mobile-phone-users-worldwide/.

[9] Agangiba W.A., Agangiba M.A. (2013). Mobile Solution for Metropolitan Crime Detection and Reporting. J. Emerg. Trends Comput. Inf. Sci..

[10] Walters C.H. Incident Detection Primarily by Cellular Phones: An Evaluation of a System for Dallas. Proceedings of the 78th Transportation Research Board Annual Meeting.

[11] Hellstrom J. (2010). Mobile Technology as a Means to Fight Corruption in East Africa. Increasing Transparency & Fighting Corruption Through ICT. http://www.diva-portal.org/smash/record.jsf?pid=diva2%3A955404&dswid=7959.

[12] Tundis A., Uzair M., Mühlhäuser M. Human Physical Status detection related to Danger Situations based on Smartwatch and Smartphone. Proceedings of the 19th IFIP Networking 2020 Conference.

[13] Fabito B.S., Balahadia F.F., Cabatlao J.D.N. AppLERT: A mobile application for incident and disaster notification for Metro Manila. Proceedings of the 2016 IEEE Region 10 Symposium (TENSYMP) 2016.

[14] Cisco IBSG The Internet of Things—How the Next Evolution of the Internet Is Changing Everything. https://www.cisco.com/c/dam/en_us/about/ac79/docs/innov/IoT_IBSG_0411FINAL.pdf.

[15] Ashton K. (2009). That ‘internet of things’ thing. RFID J..

[16] Voigt T., Rohner C. What Is the Internet of Things: An Introduction. https://ieeexplore.ieee.org/courses/details/EDP486.

[17] Gomez A.K., Bajaj S. Challenges of Testing Complex Internet of Things (IoT) Devices and Systems. Proceedings of the 11th International Conference on Knowledge and Systems Engineering (KSE).

[18] Ivan I., Ciurea C. Quality Characteristics of Collaborative Systems. Proceedings of the 2009 Second International Conferences on Advances in Computer-Human Interactions.

[19] Chung H.M. Toward implementing a mobile collaborative system. Proceedings of the 2012 International Conference on Systems and Informatics (ICSAI2012).

[20] Fling B. (2009). Mobile Design and Development: Practical Concepts and Techniques for Creating Mobile Sites and Web Apps.

[21] Hu Y., Zhang X., Hu L. A fast approach to enable mobile apps with Geo-Location logging and reporting. Proceedings of the 2015 23rd International Conference on Geoinformatics.

[22] Fernando M.C.G. STREETWATCH: A Mobile Application for Street Crime Incident Avoidance and Safety Solution. Proceedings of the TENCON 2015—2015 IEEE Region 10 Conference.

[23] Jakkhupan W., Klaypaksee P. A web-based criminal record system using mobile device: A case study of Hat Yai municipality. Proceedings of the 2014 IEEE Asia Pacific Conference on Wireless and Mobile.

[24] De Guzman J.B., de Guzman R.C.C., Ado R.G. (2014). Mobile Emergency Response Application Using Geolocation for Command Centers. Int. J. Comput. Commun. Eng..

[25] Romano M., Onorati T., Aedo I., Diaz P. (2016). Designing Mobile Applications for Emergency Response: Citizens Acting as Human Sensors. Sensors.

[26] Edillo S.B., Garrote P.J.E., Domingo L.C.C., Malapit A.G., Fabito B.S. A mobile based emergency reporting application for the Philippine National Police Emergency Hotline 911: A case for the development of i911. Proceedings of the 2017 Tenth International Conference on Mobile Computing and Ubiquitous Network (ICMU).

[27] Bhutto Z., Dahri K., Lakho I., Memon S. Social Video Streaming (SVS): A prototype application for street crime reporting. Proceedings of the 2015 International Conference on Cyber Situational Awareness, Data Analytics and Assessment (CyberSA).

[28] (2019). Red Panic Button. http://redpanicbutton.com/.

[29] (2019). SafeTrek. https://www.safetrekapp.com/.

[30] (2019). Bsafe. https://getbsafe.com/.

[31] Droid Life (2019). Android Emergency Information. http://www.droid-life.com/2016/03/09/android-n-lets-you-add-personal-emergency-info-to-your-lock-screen/.

[32] 9to5Mac (2019). Apple Medical ID. https://9to5mac.com/2014/09/21/medical-idios8/.

[33] Sp0n Inc (2019). Citizen. https://itunes.apple.com/us/app/citizen/id1039889567?mt=8;2https://play.google.com/store/apps/details?id=sp0n.citizen&hl=enUS.

[34] Warn-App NINA. https://www.bbk.bund.de/DE/NINA/Warn-App_NINA_node.html.

[35] TS Systems (2019). Crime Mapping. https://www.crimemapping.com/.

[36] PDC Global (2019). Disaster Alert. https://www.pdc.org/apps/disaster-alert/.

[37] Mateusiak J. (2019). Safety Guardian. https://play.google.com/store/apps/details?id=com.jakubmateusiak.safetyguardian&hl=de.

[38] (2020). Watch over Me. http://watchovermeapp.com/.

[39] (2020). Lifeline Response. http://llresponse.com/.

[40] Guardian Project (2019). Panickit Framework. https://guardianproject.info/tag/panic/.

[41] (2019). App Armor. http://www.apparmor.com/.

[42] Tundis A., Huber F., Jäger B., Daubert J., Vasilomanolakis E., Mühlhäuser M. (2018). Challenges and available solutions against organized cyber-crime and terrorist networks. WIT Transactions on The Built Environment.

[43] Tundis A., Mühlhäuser M. (2019). The role of Information and Communication Technology (ICT) in modern criminal organizations. Organized Crime and Terrorist Networks.

[44] Jirovský V., Pastorek A., Mühlhäuser M., Tundis A. Cybercrime and Organized Crime. Proceedings of the 13th Proceedings of the 13th International Conference on Availability, Reliability and Security (ARES 2018).

[45] Falkoff J. (2020). 4 Things you Need to Understand about Edge Computing. https://venturebeat.com/2020/03/29/4-things-you-need-to-understand-about-edge-computing/.

[46] (2020). Ericsson—Thinking of Deploying Edge Computing? Here Are Four Approaches. https://www.ericsson.com/en/blog/2020/2/thinking-of-deploying-edge-computing-here-are-four-approaches.

[47] Javascript. https://developer.mozilla.org/en-US/docs/Web/JavaScript/.

[48] Node.js. https://nodejs.org/en/download/.

[49] Wexler J. (2019). Get Programming with Node.js.

[50] Firebase. https://firebase.google.com/.

[51] Firebase and Flutter. https://flutter.dev/docs/development/data-and-backend/firebase.

[52] Firebase’s Scalable Backend Makes It ‘10 Times Easier’ to Build Apps. https://venturebeat.com/2013/02/13/firebases-backend-makes-it-ten-times-easier-to-build-apps/.

[53] Dincer A., Uraz B. (2013). Google Maps JavaScript API Cookbook.

[54] Griffith C. (2017). Mobile App Development with Ionic: Cross-Platform Apps with Ionic, Angular, and Cordova.

[55] Cheng F. (2018). Build Mobile Apps with Ionic 4 and Firebase: Hybrid Mobile App Development.

[56] Eichorn J. (2008). Understanding AJAX: Using JavaScript to Create Rich Internet Applications.

[57] Ajax. https://www.w3schools.com/jquery/jquery_ref_ajax.asp.

[58] (2020). Average Police Response Time by Category of Crimes. https://www.creditdonkey.com/average-police-response-time.html.

[59] (2020). Average Police Response Time. https://www.asecurelife.com/average-police-response-time/.

[60] (2020). Numbeo. https://it.numbeo.com/.

